# Differential sensitivity of the MMSE and MoCA for staging and longitudinal cognitive change in Huntington's disease

**DOI:** 10.1177/18796397261461641

**Published:** 2026-06-29

**Authors:** Krisha Bagga, Ben Shifflett, Anvit Sidhu, Paul E Gilbert, Jody Corey-Bloom

**Affiliations:** 1 Department of Neuroscience, 8784 UC San Diego, La Jolla, CA, USA; 2 Department of Psychology, 7117 San Diego State University, San Diego, CA, USA

**Keywords:** MMSE, MoCA, Huntington's disease, HD-ISS, cognitive testing

## Abstract

**Objective:**

To compare the sensitivity of the Mini-Mental State Examination (MMSE) and the Montreal Cognitive Assessment (MoCA) for detecting cognitive change in Huntington's disease (HD) subjects stratified by baseline performance quartiles and the Huntington's Disease Integrated Staging System (HD-ISS).

**Methods:**

Participants (age 25–65; CAG 40–50) from an observational cohort completed MMSE and MoCA at baseline, with follow-up visits at least six months after baseline, and observations truncated at five years.

We analyzed the full cohort (n = 246) and an HD-ISS–staged subset (n = 132). Longitudinal change was evaluated using mixed-effects models, adjusted for baseline age, gender, education, CAG, and age × CAG interaction. Baseline discrimination between HD-ISS stages was assessed with ROC curves and AUC comparisons.

**Results:**

Annual decline was nearly identical for MMSE and MoCA (–0.334 vs. −0.331). Adjusted quartile analyses revealed differential sensitivity: MoCA showed greater decline in lower and higher performance levels (Quartiles 1, 3, and 4), while MMSE was most sensitive at mid-range (Quartile 2). In HD-ISS analyses, MMSE demonstrated more consistent decline across stages, particularly Stages 1 and 3, indicating sensitivity to early and moderate disease phases. Baseline ROC analyses favored MoCA for stage discrimination (Stage 0 vs. all: AUC 0.789 vs. 0.686, p = 0.011).

**Conclusion:**

The MMSE and MoCA exhibit complementary strengths. MoCA demonstrates superior sensitivity for baseline staging and early disease detection, while MMSE better captures longitudinal decline. These findings suggest that combined use of both instruments may provide a more complete assessment of cognitive progression in HD.

## Introduction

Cognitive screening is essential in Huntington's disease (HD), yet the optimal instrument for longitudinal assessment remains debated. Cross-sectional evidence indicates that the Montreal Cognitive Assessment (MoCA) is more sensitive than the Mini-Mental State Examination (MMSE), particularly among individuals with mild impairment.^1–4^ This advantage is generally attributed to the MoCA's broader scoring range and reduced ceiling effects, which enhace discrimination across early disease stages.

Longitudinal findings are less consistent. Both the MoCA and MMSE detect short-term cognitive decline in HD,^5^ but the MMSE may be more informative in moderate-to-severe stages when impairment is pronounced.^6^ However, few studies have directly compared these measures longitudinally, leaving uncertainty regarding their relative sensitivity across disease stages.

This study represents the first head-to-head longitudinal comparison of the MoCA and MMSE across the full HD spectrum. By examining sensitivity to change in participants stratified by baseline performance quartiles and HD-ISS stage, this work clarifies how each instrument performs across disease stages, with implications for outcome measure selection in HD clinical trials and observational studies.

## Methods

### Participants

This study was approved by the University of California, San Diego (UCSD) Institutional Review Board (IRB) Committee (IRB Protocol #170038), in accordance with the requirements of the Code of Federal Regulations on the Protection of Human Subjects. All participants provided written informed consent prior to participating.

The Huntington's disease (HD) dataset comprised 344 participants aged 25–65 years at baseline with CAG repeat lengths between 40 and 50, each with available Mini-Mental State Examination (MMSE) and Montreal Cognitive Assessment (MoCA) data from the same visit. These age and CAG parameters were chosen to capture individuals within a mid-range risk and onset window. Of these, 246 had longitudinal MMSE and MoCA data, with follow-up visits occurring at least six months after baseline, and observations truncated at five years of follow-up.

A secondary dataset was defined by further restricting the sample to individuals with baseline PIN-derived Huntington's Disease Integrated Staging System (HD-ISS) data. A participant's PIN score was calculated using the previously published and validated formula:^7^

$$\begin{array}{rl} \mathrm{PIN} & =((51\times \mathrm{TMS}+(-34)\times \mathrm{SDMT}+7\times \mathrm{Age}\;\mathrm{at}\;\mathrm{visit}\\ & \times \;(\mathrm{CAG}-34))-883)/1044. \end{array}$$

Participants with a PIN score ≤ −0.34 were classified as Stage 0; with scores > −0.34–0.60 as Stage 1; scores of >0.60–2.31 as Stage 2; and scores greater than 2.31 as Stage 3. These thresholds were originally established to maximize separation between HD-ISS stages across all available PIN score options.^8^ The subset with baseline PIN-derived HD-ISS data included 188 eligible participants, which was reduced to 132 after applying the six-month follow-up criterion. As in the primary dataset, follow-up was truncated at five years due to the limited number of participants with longer-term data.

### Baseline score comparisons

Baseline MMSE and MoCA scores were correlated with neuropsychological and functional measures using Spearman's rank correlation to account for non-normal score distributions. Given the MoCA's greater emphasis on executive function, we anticipated differences between the two measures and examined these as a function of HD-ISS stage. The ability of baseline scores to discriminate HD-ISS stage was further evaluated using receiver operating characteristic (ROC) analysis, with area under the curve (AUC) values compared using DeLong's test.

### Statistical analysis

To evaluate longitudinal change across performance levels, participants were grouped by quartiles of baseline MMSE and MoCA scores, derived from their score distributions. In the subset with baseline HD-ISS staging, HD-ISS stage was also used as a grouping variable, given its established relationship with disease progression and cognition. Longitudinal change in MMSE and MoCA was modeled using mixed-effects analyses with random intercepts and slopes to account for within-subject correlations and individual variability in rate of change. Annual change was estimated using both unadjusted models (results shown in supplement Figures S1 and S2) and models adjusted for baseline age, gender, education, CAG repeat length, and the age × CAG interaction.

## Results

### Participant characteristics and follow-up

Baseline demographic and clinical characteristics for participants with both MMSE and MoCA data are presented in Table 1. The mean follow-up duration within five years of baseline was 2.24 ± 1.29 years. A total of 103 participants (42.3%) completed a visit between three and four years post-baseline.

**Table 1.** Baseline demographics and MMSE/MoCA scores: overall and by quartile.

Variable	n	x	s	Min	Max
Female Sex	132 (53.7%)				
Baseline Age	246	46.1	10.4	25	64
Education	246	14.7	2.9	3	22
CAG	246	43.2	2.4	40	50
Baseline MMSE QT1	73	21.3	2.6	13	24
Baseline MMSE QT2	71	26.2	0.8	25	27
Baseline MMSE QT3	77	28.6	0.5	28	29
Baseline MMSE QT4	25	30.0	0.0	30	30
MMSE Overall	246	25.9	3.6	13	30
Baseline MoCA QT1	65	15.4	3.6	6	20
Baseline MoCA QT2	62	22.6	1.2	21	24
Baseline MoCA QT3	72	26.1	0.8	25	27
Baseline MoCA QT4	47	28.7	0.8	28	30
MoCA Overall	246	22.9	5.3	6	30

x is the mean. s is the standard deviation. MMSE= Mini Mental State Examination; MoCA= Montreal Cognitive Assessment; QT= quartile.

Supplementary Figure 3 (Figure S3) illustrates time from baseline by visit number, showing mean intervals of approximately one year for visits 2–4, with increasing variability thereafter reflecting differences in follow-up schedules. The mean change from baseline to the last available visit was −1.13 points for both the MMSE and MoCA, with no significant difference between measures.

### Quartile analyses

Baseline MMSE and MoCA scores were divided into quartiles based on their score distributions: Quartile 1 = lowest 25%, Quartile 2 = 25–50%, Quartile 3 = 50–75%, and Quartile 4 = highest 25%. Cut points were 24, 27, and 29 for MMSE and 20, 24, and 27 for MoCA (Table 1). A ceiling effect was evident for the MMSE, with all Quartile 4 participants achieving the maximum score of 30. In contrast, MoCA quartiles displayed a more even score distribution across the range.

### Adjusted models

After adjusting for baseline age, gender, education, CAG repeat length, and the age × CAG interaction, annual decline was nearly identical for MMSE (β = –0.334) and MoCA (β = –0.331) (Table 2). All MMSE quartiles demonstrated significant decline, with the steepest slope observed in Quartile 2 (β = –0.457, p < 0.001), representing a shift from Quartile 3 in the unadjusted models. For the MoCA, declines were significant in Quartiles 1, 3, and 4. In Quartile 1, the rate of decline was steeper for MoCA (β = –0.508, p = 0.004) than for the MMSE (β = –0.313, p = 0.043). The absence of decline in MoCA Quartile 2 suggests heterogeneity in sensitivity across baseline performance levels and underscores the complementary strengths of the two measures.

**Table 2.** MMSE and MoCA annual decline: overall and by quartile (adjusted).

	MMSE			MoCA		
	Estimate	Std. Error	p-value	Estimate	Std. Error	p-value
QT1 Time	−0.313	0.151	0.043	−0.508	0.168	0.004
QT2 Time	−0.457	0.121	0.001	−0.121	0.126	0.341
QT3 Time	−0.332	0.088	0.000	−0.450	0.132	0.001
QT4 Time	−0.355	0.091	0.001	−0.389	0.089	0.000
Overall Time	−0.334	0.055	0.000	−0.331	0.062	0.000

Abbreviations: MMSE: Mini Mental State Examination; MoCA: Montreal Cognitive Assessment; QT: Quartile.

### MMSE/MoCA versus cognitive/functional measures

At baseline, the MMSE and MoCA were highly correlated (r = 0.80, p < .0001; n = 246; Table 3). In the subset with additional neuropsychological data (n = 96), both measures were significantly associated with functional capacity as measured by the Total Functional Capacity (TFC) scale (r = 0.58, p < .0001 for both MMSE and MoCA) and with performance across multiple cognitive domains. Across individual tests, MoCA correlations were numerically higher than those for the MMSE—for example, category verbal fluency (r = 0.55 vs. 0.48), phonemic fluency (0.63 vs. 0.56), Stroop interference (0.58 vs. 0.48), and Trails A (–0.56 vs. −0.45) and B (–0.67 vs. −0.61). However, Steiger's tests indicated that none of the MoCA–MMSE correlation differences reached statistical significance (all p > .33).

**Table 3.** MMSE and MoCA spearman correlations with cognitive/functional items.

		MMSE		MoCA		Steigler test	
	n	Corr	p.val	Corr	p.val	z	p.val
MMSE ∼ MoCA	246	0.80	<0.0001	0.80	<0.0001	0.00	1.0000
TFC	96	0.58	<0.0001	0.58	<0.0001	0.00	1.0000
Categorical Verbal Fluency	96	0.48	<0.0001	0.55	<0.0001	0.65	0.5154
Stroop Interference	89	0.48	<0.0001	0.58	<0.0001	0.91	0.3604
Trails A	89	−0.45	<0.0001	−0.56	<0.0001	0.97	0.3314
Trails B	89	−0.61	<0.0001	−0.67	<0.0001	0.67	0.5043
FAS Total	89	0.56	<0.0001	0.63	<0.0001	0.71	0.4764

Abbreviations: MMSE: Mini Mental State Examination; MoCA: Montreal Cognitive Assessment; TFC: Total Functional Capacity.

### HD-ISS stage analyses

#### Participant characteristics and follow-up

Baseline demographics and clinical characteristics for participants with MMSE/MoCA data and PIN-derived HD-ISS staging are shown in Table 4. The mean follow-up duration within five years of baseline was 2.45 ± 1.25 years. A total of 59 participants (44.7%) completed a visit between three and four years post-baseline.

**Table 4.** Baseline demographics and MMSE/MoCA scores: overall and by HD-ISS stage.

Variable	n	x	s	Min	Max
Female Sex	71 (53.8)				
Baseline Age	132	45.5	10.4	25	65
Education	132	14.9	2.9	3	22
CAG	132	43.1	2.5	40	50
Baseline MMSE Stage 0	27	27.8	1.6	24	30
Baseline MMSE Stage 1	25	28.2	1.2	26	30
Baseline MMSE Stage 2	37	26.5	2.4	21	30
Baseline MMSE Stage 3	43	23.7	3.7	15	29
MMSE Overall	132	26.2	3.2	15	30
Baseline MoCA Stage 1	27	27.3	2.1	21	30
Baseline MoCA Stage 2	25	26.7	2.0	23	30
Baseline MoCA Stage 3	37	24.4	2.6	19	29
Baseline MoCA Stage 4	43	20.0	4.9	10	28
MoCA Overall	132	24.0	4.5	10	30

x is the mean. s is the standard deviation. Abbreviations: MMSE: Mini Mental State Examination; MoCA: Montreal Cognitive Assessment.

Supplementary Figure 4 (Figure S4) illustrates time from baseline by visit number, showing mean intervals of approximately one year for visits 2–4, with greater variability at later visits, reflecting differences in follow-up scheduling. The mean change from first to last visit was −0.57 points for the MMSE and −0.38 points for the MoCA, a difference that was not statistically significant (p = 0.59). The mean interval between first and last visits was 3.12 years.

### Adjusted models

After adjusting for baseline age, gender, education, CAG repeat length, and the age × CAG interaction, annual declines remained comparable for the MMSE (β = –0.334, p < 0.001) and the MoCA (β = –0.331, p < 0.001) (Table 5). When stratified by HD-ISS stage, the MMSE demonstrated significant decline in both Stage 1 (β = –0.283, p = 0.039) and Stage 3 (β = –0.419, p = 0.009), whereas no HD-ISS stage showed significant decline on the MoCA. These results suggest that the MMSE may offer greater sensitivity than the MoCA for detecting longitudinal change across HD-ISS stages, particularly in manifest disease.

**Table 5.** MMSE and MoCA annual decline: overall and by HD-ISS stage (adjusted).

	MMSE			MoCA		
	Estimate	Std.Error	p-value	Estimate	Std. Error	p-value
Stage 0 Time	0.101	0.074	0.179	0.120	0.107	0.265
Stage 1 Time	−0.283	0.128	0.039	−0.137	0.130	0.306
Stage 2 Time	−0.039	0.154	0.799	−0.203	0.161	0.216
Stage 3 Time	−0.419	0.055	0.009	−0.200	0.162	0.220
Overall Decline	−0.334	0.055	0.000	−0.331	0.062	0.000

### PIN-derived HD-ISS dataset: ROC curve analysis

ROC analyses based on MMSE quartiles were omitted, as classifying MMSE quartiles with the MMSE would be tautological. Instead, we focused on ROC analyses using the independent PIN-derived HD-ISS staging system, in conjunction with longitudinal decline analyses, to provide a more robust assessment of the relative utility of the MMSE and MoCA (Figure 1).

**Figure 1.**
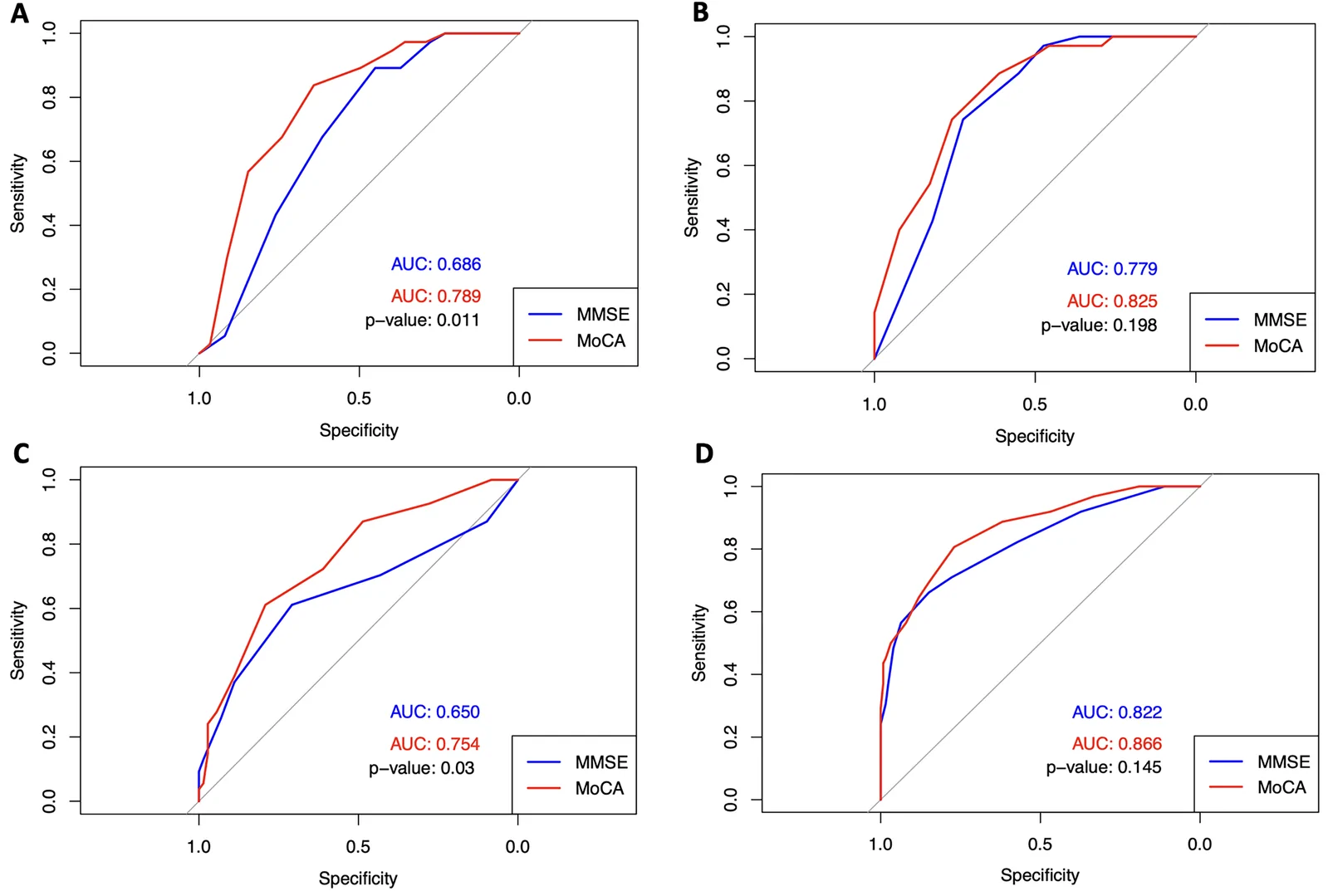
ROC curves for HD-ISS stage classification. A= PIN-derived ISS Stage 0 v 1–3 Classification; B= PIN-derived ISS Stage 1 v. 2–3; C= PIN-derived Stage 2 v 0–1 Classification; D= Pin-derived ISS Stage 3 v. 0–2 Classification.

The MoCA demonstrated superior discrimination relative to the MMSE for distinguishing Stage 0 from all other stages (AUC = 0.789 vs. 0.686, p = 0.011) and for distinguishing Stage 2 from Stages 0–1 (AUC = 0.754 vs. 0.650, p = 0.030). In contrast, discrimination between Stage 1 versus Stages 2–3 and Stage 3 versus Stages 0–2 did not differ significantly between measures. (Figure 1)

## Discussion

To our knowledge, this is the first direct, longitudinal comparison of the MMSE and MoCA in a large well-characterized HD cohort using complementary analytic approaches—HD-ISS stage stratification, longitudinal mixed-effects modeling, and ROC analysis. Although overall annual decline across the full sample was similar for the two instruments, stratified analyses revealed meaningful differences in how each measure performed across the disease spectrum.

At baseline, MMSE scores showed a pronounced ceiling effect among higher-functioning participants, whereas MoCA scores were more evenly distributed, supporting detection of subtle cognitive deficits. In ROC analyses against PIN-derived HD-ISS staging classifications, MoCA more accurately discriminated disease stages—particularly distinguishing Stage 0 from all others, and Stage 2 from Stages 0–1—consistent with its broader coverage of executive and attentional domains and reduced ceiling effects. Because HD-ISS staging was derived independently of MMSE/MoCA item content, these ROC findings are not attributable to score circularity.

In HD-ISS stage-stratified longitudinal models, the MMSE showed more consistent and statistically signifcant decline –most notably in Stages 1 and 3– whereas MoCA declines were less stage-specific and did not reach significance. This pattern suggests that the MMSE may be more effective for tracking disease progression once manifest HD is present, while the MoCA provides greater sensitivity for early-stage detection and cross-sectional classification. These findings refine earlier reports of cognitive decline trajectories in HD^5^ and align with prior work indicating that the MMSE retains longitudinal utility in moderate-to-severe stages despite ceiling constraints in early disease.^6^

Consistent with prior reports by Bezdicek et al., Gluhm et al., and Rosca & Simu,^1–3^ we found the MoCA to be more sensitive than the MMSE in premanifest and early HD, likely due to its wider score range and greater emphasis on executive processes. Our results extend these findings by showing that MoCA's advantage at baseline persists when participants are stratified by HD-ISS stage, while MMSE better captures longitudinal decline once cognitive impairment is established.

Strengths of our study include a relatively large HD cohort with repeated MMSE and MoCA assessments over up to five years, stratified analyses by both baseline performance quartiles and HD-ISS stage, and mixed-effects modeling with covariate adjustment for age, sex, education, CAG repeat length, and the age × CAG interaction.

Nonetheless, several limitations should be acknowledged. Follow-up schedules varied due to the observational nature of the dataset, introducing potential variability in measurement intervals. Although the cohort was large for a single-site HD study, sample size remained modest for stratified analyses, particularly among Stage 0 participants with limited follow-up. Few participants were beyond three years, restricting inference about longer-term cognitive trajectories. Unmeasured factors (e.g., comorbidities, medications, mood) may have influenced performance despite covariate adjustment. In addition, neither MMSE nor MoCA is HD-specific; both may underestimate subtle decline in domains such as social cognition or psychomotor speed. Finally, because inclusion was limited to participants aged 25–65 years with CAG repeat lengths between 40 and 50, results may not generalize to very early or late-onset HD cases.

For HD research and clinical trials, the MoCA appears preferable for early-stage detection and baseline stratification, whereas the MMSE may be better suited for tracking longitudinal decline once manifest disease is established. Future studies should test these roles in larger, multi-site cohorts (e.g., Enroll-HD), that allow greater sample sizes and extended follow-up. Comparative analyses incorporating additional cognitive measures, including HD-specific instruments, will also be important to contextualize the relative sensitivity and clinical utility of the MMSE and MoCA across disease stages.

### Conclusion

In this large longitudinal HD cohort, both the MMSE and the MoCA detected significant overall cognitive decline, though their relative strengths differed by analytic approach. The MoCA demonstrated superior baseline discrimination of HD-ISS stages, likely reflecting its broader score range and emphasis on executive function, whereas the MMSE showed more consistent sensitivity to longitudinal change in stage-stratified analyses. These complementary strengths suggest that cognitive test selection in HD research should be guided by study objectives—favoring the MoCA for early-stage detection and cross-sectional classification, and the MMSE for monitoring decline over time. Combining both measures may offer the most comprehensive assessment of cognitive change across the HD spectrum.

## Supplemental Material

sj-docx-1-hun-10.1177_18796397261461641 - Supplemental material for Differential sensitivity of the MMSE and MoCA for staging and longitudinal cognitive change in Huntington's disease Supplemental material, sj-docx-1-hun-10.1177_18796397261461641 for Differential sensitivity of the MMSE and MoCA for staging and longitudinal cognitive change in Huntington's disease by Krisha Bagga, Ben Shifflett, Anvit Sidhu, Paul E Gilbert and Jody Corey-Bloom in Journal of Huntington's Disease

sj-jpg-2-hun-10.1177_18796397261461641 - Supplemental material for Differential sensitivity of the MMSE and MoCA for staging and longitudinal cognitive change in Huntington's disease Supplemental material, sj-jpg-2-hun-10.1177_18796397261461641 for Differential sensitivity of the MMSE and MoCA for staging and longitudinal cognitive change in Huntington's disease by Krisha Bagga, Ben Shifflett, Anvit Sidhu, Paul E Gilbert and Jody Corey-Bloom in Journal of Huntington's Disease

## Data Availability

The datasets analyzed during the current study are available from the corresponding author on request.
