# Supplementary material for: Differential sensitivity of the MMSE and MoCA for staging and longitudinal cognitive change in Huntington's disease
Source: J Huntingtons Dis. 2026 Jun 29;15(4):635–40. doi: 10.1177/18796397261461641 (PMC13768561; doi:10.1177/18796397261461641)
Supplement: Supplementary file 1 [file sj-docx-1-hun-10.1177_18796397261461641.docx]

**SUPPLEMENT**

Figure 1S. Boxplot: Years from Baseline by Visit Number

Figure 2S: Boxplot: Years from Baseline by Visit Number
